# Clinical validation of myOSLchip: A beryllium oxide optically stimulated luminescent dosimeter (OSLD) system in radiotherapy dosimetry

**DOI:** 10.1002/acm2.70094

**Published:** 2025-04-24

**Authors:** Heath Davis, Jeffrey V. Siebers, Krishni Wijesooriya, Matt Mistro

**Affiliations:** ^1^ Department of Radiation Oncology University of Virginia Charlottesville Virginia USA

**Keywords:** dosimetry, myOSLchip, OSLD

## Abstract

In radiation oncology, inter‐fractional dosimetry using optically stimulated luminescent detectors (OSLDs) ensures accurate plan delivery and patient safety. RadPro International GmbH's myOLSchip system, featuring a beryllium oxide (BeO) OSL dosimeter, reader, and eraser, was characterized and calibrated with a Varian Truebeam for in‐vivo dosimetry following AAPM TG‐191 guidelines. The BeO detectors demonstrated good dose linearity and repeatability across multiple exposures and erasure cycles, aligning with the manufacturer's stated accuracy and precision.

## 1 INTRODUCTION

Optically stimulated luminescent detectors (OSLDs) have been widely used in the medical physics community due to their ease of use, including the ability to be placed in arbitrary locations, signal stability, response linearity, and rapidly read out in the clinic. Like TLDs, OSLDs store charge when radiation interacts with material, and upon stimulation, the light produced is proportional to the dose received by the device allowing for accurate dosimetry when properly calibrated. The Landauer nanoDot is one such OSLD comprised of Al_2_O_3_:C and was widely popular in clinical radiotherapy as an in‐vivo dosimeter due to its small form factor, light‐tight encapsulation, and low cost per dosimeter.^1^, ^2^ However, the nanoDot is no longer available, leaving users in need of a replacement system.

This study characterizes myOSLchip, an OSLD like the nanoDot, with a small form factor and light‐tight encapsulation, with a low cost per dosimeter. The comparable size and performance of the nanoDot and myOSLchip would allow clinics familiar with the former system to commission the new dosimeter without major changes to their established workflows. Table 1 shows a comparison of the nanoDot and myOSLchip. AAPM TG191 references many publications that studied the nanoDot system.^3^ This work investigates the dosimetric characteristics of myOSLchip, a beryllium oxide (BeO) based OSLD system. Linearity, accuracy, and other properties are explored in a clinical setting for therapeutic MV photon and electron beam dose measurements.^4^, ^5^

**TABLE 1:** A comparison of the nanoDot and myOSLchip dosimeters.

Name	Material	*ρ* (g/cm^3^)	Z_eff_	Accuracy	Package dimension (mm)	Sensitive area (mm)
nanoDot	Al_2_O_3_:C	3.95	11.28	±10%, screened ± 5.5%	10 × 10 × 2	Ø 4 × 0.3
myOSLchip	BeO	2.85	7.21	±5%	9.5 × 10 × 2	4.7 × 4.7 × 0.5

## 2 METHODS AND MATERIALS

### 2.1 Dosimeter

**FIGURE 1:**
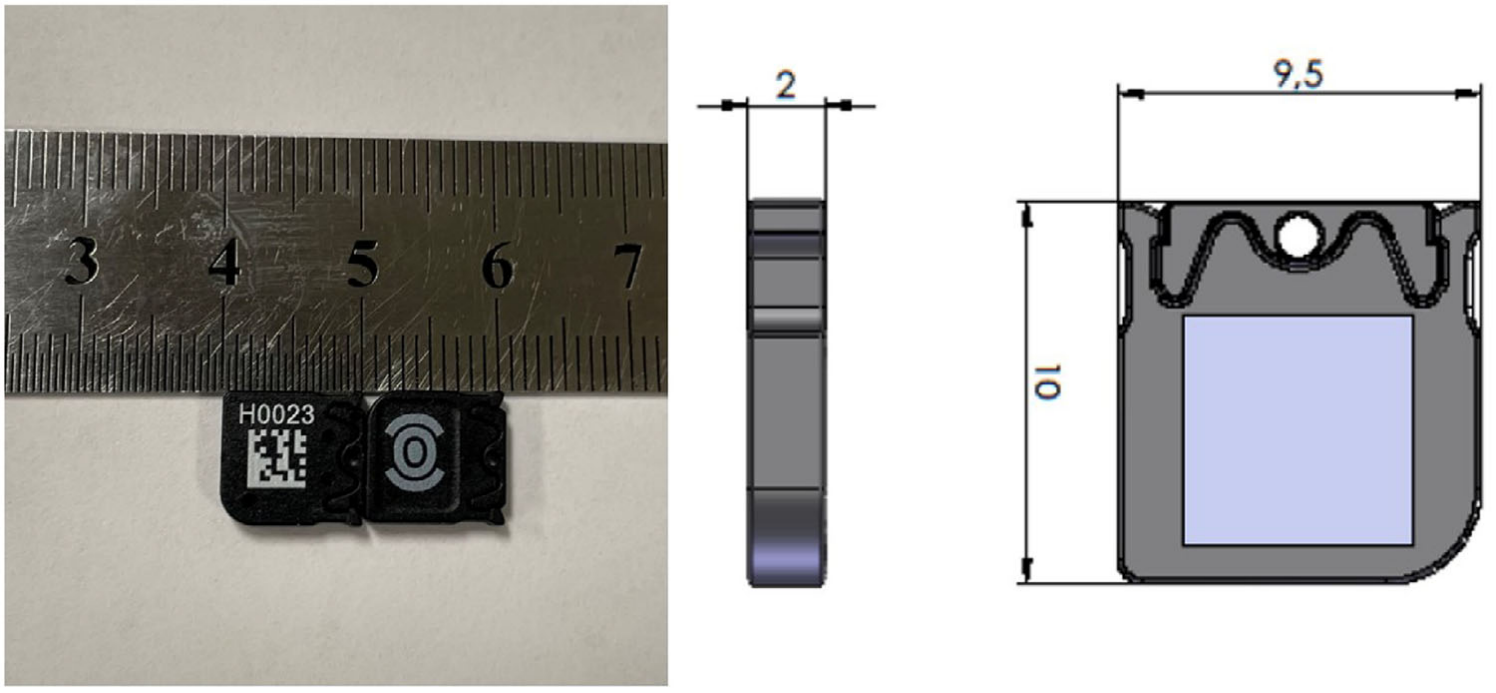
The left image shows the front and back sides of the myOSLchip with a 2‐D bar code on with a cm ruler for scale. Right shows a technical drawing from the manual with the dimensions in mm for scale with a grey/blue box showing the location of the BeO element inside.^7^ BeO, beryllium oxide.

The myOSLchip dosimeter (Figure 1) is comprised of a 4.7 × 4.7 × 0.5 mm BeO element inside a 9.5 × 10 × 2 mm ABS (Acrylonitrile‐Butadiene‐Styrol‐Copolymer) light‐tight housing. BeO has a density of 2.85 g/cm^3^ with a Z_eff_ of 7.21.^4^, ^5^ Each chip has a 4‐digit number and 2‐D barcode printed on the housing for identification visually as well as by the reader which allows the system or user to keep track of the read‐out history of each OSL, including any correction factors associated with each device. The BeO element is tightly fixed in a sliding tray which is opened inside the reader or erasure devices. RadPro reports a coefficient of variation less than 1% and linearity better than 1% up to 10 Sv.^6^ A delay between exposure and readout of 10 min is recommended to allow shallow trap emission and signal stability. For high‐dose applications, RadPro suggests a maximum single dose of 10 000 mGy, a lifetime dose of 5,000,000 mGy, and a maximum accumulated bleaching time of 10 000 s.^7^

### 2.2 Reader

**FIGURE 2:**
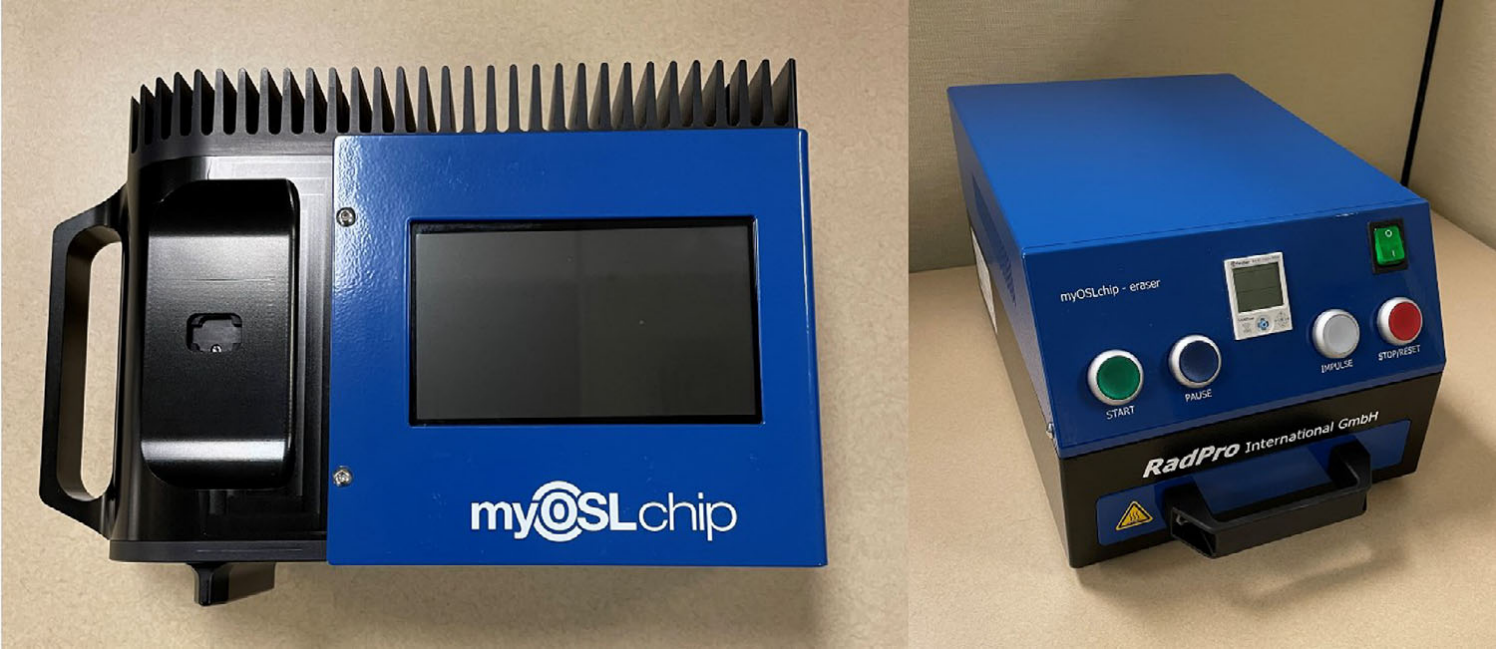
Left image shows the myOSLchip reader with the scalloped tray and slot for the dosimeter. The right image shows the high‐power LED eraser with a drawer capable of holding 48 dosimeters with an adjustable erasing time of up to 120 min.

The myOSLchip reader shown in Figure 2 is comprised of a Peltier element, photomultiplier tube, and readout electronics with an LCD screen. There is a 100 mW read LED for stimulation and a 1 W erase LED with 460 nm wavelength light. The excitation spectra of BeO is a broad peak from 420–550 nm with a maximum of 435 nm.^8^ The Peltier element stabilizes the PMT temperature at 25°C for a consistent thermal response during light collection. Readings are performed by placing the chip in the scalloped tray with the 2‐D barcode facing outward and the tray‐opening pin in the corresponding hole on the chip. By rotating the mechanism clockwise 90  into the read position, the device opens the housing, stimulates the element, and provides the number counts detected on the screen. Up to 100 dosimeters and 500 data readings can be stored directly on the device. A computer running the myOSLchip software can store more than 500 data points. The stimulation pulse length is 0.2 s with a 0.03 s delay followed by a 0.001 s read time.^7^ Rotating the mechanism an additional 45° clockwise to the erase position will expose the chip to the built‐in erase LED for 10 s if clearing low dose signal is desired. Note, in this study, the on‐reader eraser was not used.

### 2.3 Eraser

The myOSLeraser is a standalone device that enables re‐use of myOSL dosimeters. The eraser holds up to 48 myOSLchips in machined slots in a tray. A lever must be actuated when placing the tray into the eraser to open all of the chips, exposing the BeO elements to the erase LEDs. The user can select any erasure time in minutes up to 120 min which is sufficient to reduce the residual counts from a 10.5 Gy exposure (240,000 counts) to levels less than 2 mGy (< 50 counts). An unexposed dosimeter has a background reading of 20–40 counts. The standalone eraser is necessary for high‐dose applications due to the long erasure time needed to fully bleach the dosimeters.

### 2.4 Measurement conditions

Irradiations were performed with a Varian Truebeam Linear accelerator for both photon and electron measurements. Photon energies of 6 MV, 10 MV, and 15 MV were used as well as electrons at 6 MeV and 20 MeV. The Truebeam output was determined prior to irradiation using the machine performance check (MPC) output check which is within 0.17% of ion chamber measurements.^9^ The number of monitoring units delivered was adjusted in order to give a consistent dose to the dosimeters. For each energy, irradiations were conducted at 100 cm SSD, with the dosimeters at dmax under solid water with 6 cm of solid water for backscatter. Photons used a 10 × 10 cm field size and electrons used the 10 × 10 cm cone with a standard 10 cm square cutout.

### 2.5 Batch calibration $k_{s,i}$

We adopted the batch calibration method as defined in AAPM TG191, where all detectors from a single production run are simultaneously calibrated to establish individual calibration factors for each OSLD.^4^ Each dosimeter is an independent device that exhibits slight differences in performance due to manufacturing deviations, crystal inhomogeneities, and other defects. The independent response of each detector can be corrected to the mean of the entire sample. An initial 0.50 Gy irradiation was used to establish per‐OSLD calibration coefficients by determining the mean count of the batch, $\bar{M}$ divided by the signal of each device, $M_{i}$, as shown in Equation 1. This batch calibration value was used for each dosimeter for all following readings.

(1) $$\;k_{s,i}=\frac{\bar{M}}{M_{i}}$$

### 2.6 Signal fading

OSLD signals fade over time due to shallow traps spontaneously freeing due to thermal excitation and lattice defects. For this reason, it is recommended by the manufacturer to wait at least 10 min post‐irradiation before reading the device allowing the signal to stabilize. For most measurements in this study, a 10‐min wait time was observed post‐irradiation. A series of readouts were performed on some measurement sets to quantify the signal loss as a function of time. This quantification enables correcting myOSLchip signal for readout times from 10 min to 3 days.

### 2.7 Readout depletion

The act of stimulating the dosimeters in the readout process also results in a reduction in signal for each successive reading. Dosimetric accuracy is improved when this is corrected in each successive readout by adding in the signal reduction from each previous measurement. This factor was determined by exposing the dosimeters to a known reference dose of 0.50 Gy and repeatedly reading out the device A linear curve was fit and the slope of this line is then used to correct the signal for each successive reading for all dosimeters. With the nanoDot system, this depletion was negligible over the first 4–5 readouts, however, if this is not applied to the myOSLchip, the clinical impact would be an increasing uncertainty due to signal reduction. This uncertainty would compound with multiple uncorrected readouts.

### 2.8 Dose linearity

Dose response was evaluated with 6 MV photon irradiations from 0.50–10.50 Gy increasing non‐linearly in 0.50, 1.00, 1.50, 2.00, 2.50, and 3.00 Gy exposures. The measurement sequence was irradiation, 10‐min wait time, and either single or triple readouts for each batch, followed by irradiation of the next dose. Six irradiations, with cumulative doses up to 10.50 Gy were performed. Each batch of 10 dosimeters was then bleached using the eraser for 2 h. The whole cycle was repeated two times for each batch of detectors resulting in a dose‐response curve for 0, 1, and 2 erasure cycles.

### 2.9 Oblique incidence

Due to the asymmetric design of the dosimeter and the thin active area of BeO inside the device, we investigated the signal response as a function of the incidence angle on the device. This was tested with two different setups: the first setup utilized solid water in the same reference conditions described previously with five detectors placed at 0° face up (OSL symbol up) and five face down at 180° (barcode up). The second method used a cylindrical water phantom with a 3D printed ABS device holder at the isocenter, with 15 OSLs at each angle of 0°, 90°, and 180°.

### 2.10 Dosimetry testing

To determine the system reproducibility with all the correction factors, a test was conducted with a group of five detectors irradiated to 2.0 Gy with 6 MV photons, 1.5 cm of solid water for buildup, and 6 cm of backscatter at 100 cm SSD. A 10‐min wait time was observed, and each dosimeter was read out and corrected using all the relevant correction factors following the AAPM TG‐191 formalism.

## 3 RESULTS & DISCUSSION

### 3.1 Batch calibration

The individual response of each detector was determined for 50 dosimeters. Multiplying the counts observed in each detector by the sensitivity factor, with a range of 0.75–1.086, a mean of 1, and a standard deviation of 0.075, corrects the counts to the mean of the batch. The outliers of the batch calibration were not thrown out. As the magnitude of the signal correction increases, the size of the correction increases as does the uncertainty. By limiting the magnitude of the correction factor to within 1–2 standard deviations of the mean, the size of the correction, and therefore, the error introduced into the corrected reading, is limited.

### 3.2 Readout depletion

**FIGURE 3:**
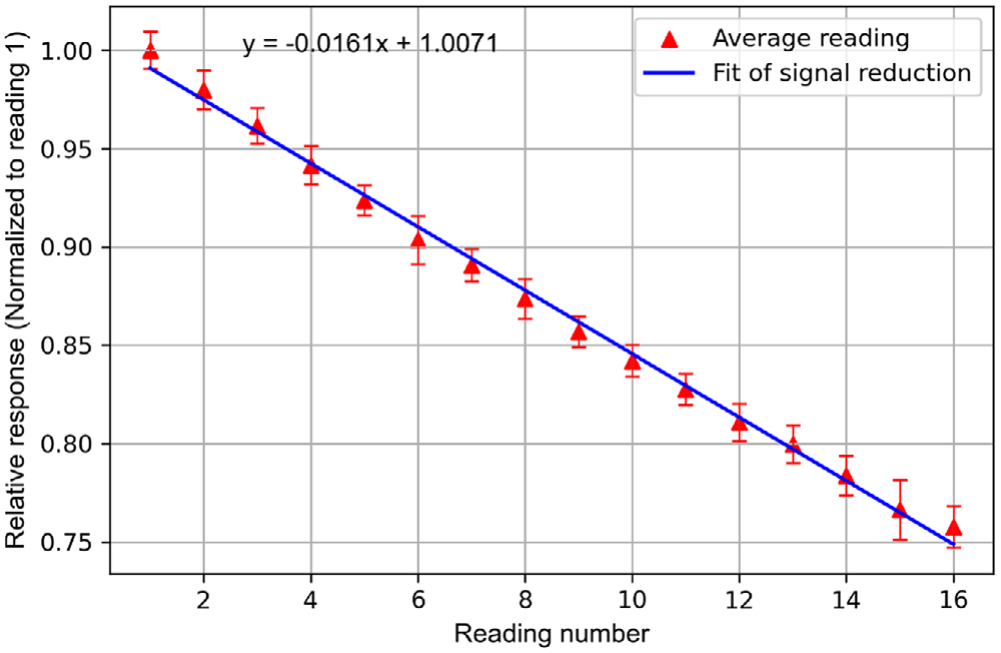
The average signal of 5 dosimeters 10 min post‐exposure. Each dosimeter was readout 16 times consecutively and their signal at each readout number was averaged among the five dosimeters. This curve was normalized to the first reading and a trend line was added to show the average signal reduction per readout.

A series of five dosimeters were read out 16 times, normalized to the first reading, and averaged showing a linear reduction in counts of 1.6% per reading as seen in Figure 3. This readout signal loss factor was used to correct the raw counts for each subsequent measurement when more than 1 reading was taken before erasure.

### 3.3 Signal fading

**FIGURE 4:**
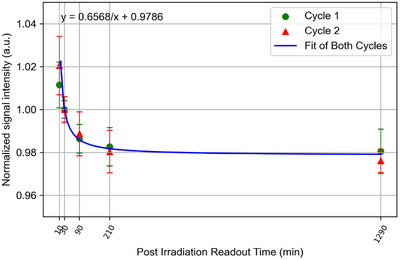
Signal versus time post 0.50 Gy irradiation for five dosimeters with cycle 1 that had no prior dose, and cycle 2 that was irradiated up to 10.5 Gy and erased by spending 2 h in the eraser. The inverse decay for the two cycles was normalized to 30 min which showed a more stable signal output than 10 min post exposure. The readout signal loss was corrected in each reading.

The signal loss follows an inverse decay and stabilizes at a 4% reduction in signal from 10 min to 20 h (Figure 4). Given that the readout of a batch of dosimeters took ∼4 min, signal fading across the group of OSLDs resulted in up to 1% uncertainty. The signal can be corrected to the 10‐min reference time post‐irradiation if the time of exposure is known using this correction curve. During analysis, it was found that 30 min post‐irradiation was a more stable reference time whereby the relative differences between the two cycles were minimized. While 10 min is considered sufficient for light output stabilization, 30 min or longer leads to more consistent time dependent light output corrections across the two cycles measured. After 3 h, the signal is stable within a few 10ths of a percent.

### 3.4 Dose linearity $k_{L}\left(D\right)$

For each reading, a 1.6% signal reduction was corrected, as was the per‐dosimeter sensitivity factor. The irradiation‐readout sequence was repeated for each dose level, yielding a cumulative OSLD dose of 10.5 Gy. One batch of detectors was readout 3 times at each dose level and one batch was readout once at each dose level. After the readout of the final dose level, the eraser was used to clear the dosimeters for 2 h. Each reading sequence was corrected using Equation 2 to normalize the dose response to the first 0.50 Gy exposure.

(2) $$k_{L}\;\left(D\right)=\frac{D_{\exp}}{M{\left(D\right)}_{\exp}}/\frac{D_{\mathrm{ref}}}{M{\left(D\right)}_{\mathrm{ref}}}$$

**FIGURE 5:**
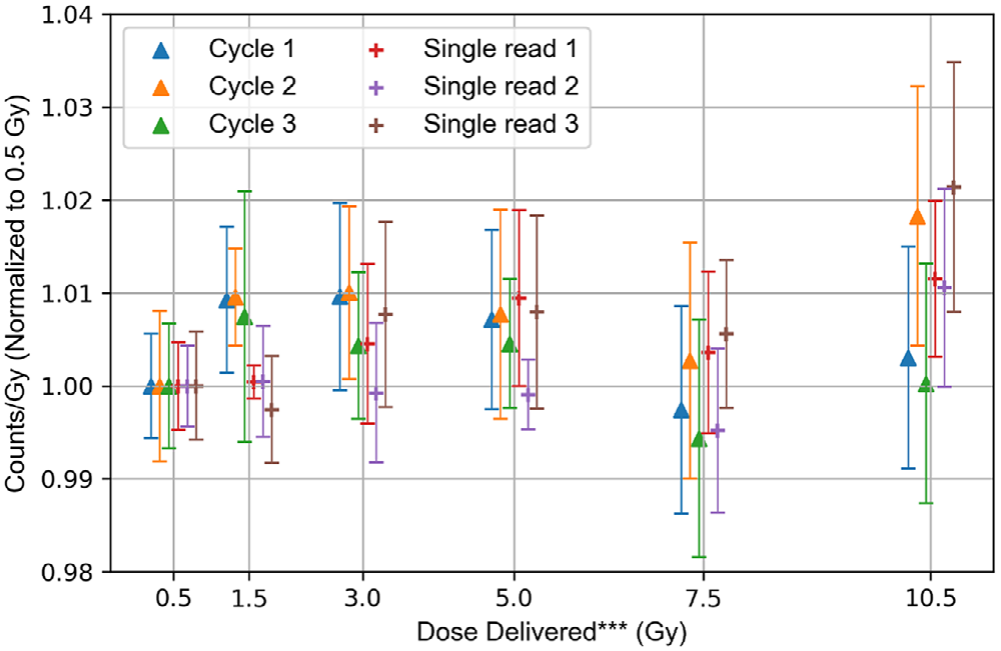
Relative response normalized to 0.50 Gy versus dose delivered for 10 dosimeters averaged, sensitivity corrected, and bleaching corrected for 3 readings per dose level from 0.5 to 10.5 Gy and 10 dosimeters with a single reading at each dose level. ***To allow clear visualization of the data and error bars, doses are offset by 0.3 Gy centered on the actual dose delivered.

*D*_ref_ is the reference dose of 0.5 Gy and M(*D*)_ref_ is the measured signal at the reference dose. *D*_exp_ and *M*(*D*)_exp_ is the dose and measured signal at each exposure level. The irradiation‐readout cycle was repeated two more times, yielding the dose response shown in Figure 5 with a consistent, linear response up to 7.5 Gy with a variation less than 1%. The variation of the single and triple readings, regardless of erasure cycle are within 1% even with the correction factor applied 27 times over the entire dose range. The non‐normalized, uncorrected counts at each dose level are within 4% of other cycles. The 10.5 Gy reading shows a variation of up to 2% and a large deviation between each reading. At 10.5 Gy the reader displays the message “High dose detected. Adjusting photosensor” when the counts exceed 200,000. This could contribute to the non‐linearity at 10.5 Gy; however we do not have evidence to support this conclusion. When building a linearity curve, another data point above 10.5 Gy would better characterize the dose response in high‐dose applications. Given the strongly linear dose per cycle up to 7.5 Gy, the *N_D,w_* calibration factor can be determined at the reference dose of 0.50 Gy to be 0.0000418 Gy/count.

### 3.5 Energy dependence

The results of the energy dependence test show no change in response for 10 and 15 MV photon beams when compared to 6 MV. Similarly, the difference between 6 MeV electrons and 20 MeV electrons is also negligible when compared to 6 MV photons. The results normalized to 6 MV and their associated uncertainties are shown in Table 2.

**TABLE 2:** Results of 10X, 15X, 6e and 20e measurements normalized to 6X.

Energy	6X	10X	15X	6e	20e
Photons	1 ± 0.026	0.992 ± 0.036	1.004 ± 0.029	–	–
Electrons	–	–	–	1.009 ± 0.028	1.01 ± 0.019

### 3.6 Oblique incidence

The first method of determining the variation between 0° and 180° in solid water resulted in a difference of 0.5% suggesting a negligible change in signal response. The second method using the 3D‐printed insert in a cylindrical water phantom resulted in a variation of response of 2.2% ± 2.26% from 0° to 180°. The uncertainty precludes a conclusive determination of angular dependence while providing an upper bound on the variation.

### 3.7 Dose calculation

To determine the reliability of the device's dose measurement in a clinical setting, a set of dosimeters were given 2.0 Gy under the same reference conditions previously stated. Dose is calculated using Equation 3 which is a modified version of the AAPM TG‐191 formalism.

(3) $$\;D_{w}=M_{\mathrm{corr}}\;\cdot N_{D,w}\cdot k_{F}\cdot k_{L}\cdot k_{Q}\cdot k_{\theta }$$

*D_w_* is the corrected dose, *M*_corr_ is the background, and individual sensitivity factor corrected counts, and *N_D,w_* is the counts to dose conversion factor. The dark current from the PMT averaged 1.2 counts per reading which can be ignored. The correction factor *k_F_* is signal fading, *k*_l_ is the dose linearity factor, *k_Q_* is the beam quality correction factor, and k_θ_ is the angular correction factor. With the error of each correction factor propagated, the OSLDs reported 1.99 ± 0.06 (3.3%) Gy. This shows that with proper characterization of each of these factors, the myOSL system can accurately report the dose delivered under the reference conditions it was calibrated for.

### 3.8 Clinical usage

The usage scenario tested in this work was for in‐field radiation measurements for external beam radiation therapy. Since the energy of out‐of‐field radiation remains Compton‐dominated, it is probable that out‐of‐field measurements that utilize in‐field calibration factors will have sufficient accuracy. However, this should be tested if high accuracy is needed.

Similarly, this work did not test the detector response in the kV energy range, or for brachytherapy applications. Until or unless energy spectrum independence is demonstrated, calibration with the beam quality of interest is warranted.

We also did not test the temperature dependence in this work. Use or storage of the detectors outside of typical clinical environmental conditions should be confirmed before use in those scenarios.^10^

## 4 CONCLUSIONS

This work has presented the myOSL system as a suitable dosimetry system in clinical radiotherapy dose range due to its dosimetric linearity with less than 1% variation up to 7.5 Gy, increasing to 2% at 10.5 Gy with the note of a potential outlier for the 10.5 Gy readings due to photosensor sensitivity changes. The readout as well as time‐dependent bleaching factors have been well characterized allowing for accurate correction factors for dose conversion. The stable count rate after multiple erasure cycles suggests the device can be erased and reused without significantly altering the dosimetric properties.

## AUTHOR CONTRIBUTIONS

All authors contributed to the study design. Heath Davis performed measurements, collected data, and drafted the initial manuscript. Jeffrey V. Siebers, Krishni Wijesooriya, and Matt Mistro guided the analysis and verified the analytical methods. Heath Davis and Matt Mistro prepared the figures. All authors contributed to the final manuscript and approved the submission.

## CONFLICT OF INTEREST STATEMENT

No conflicts of interest were identified, and all data was collected and analyzed objectively.

## ETHICS STATEMENT

This research was conducted in accordance with the highest ethical standards.

## Data Availability

Data will be made available by request from the corresponding author.
